# CEMRA in neonatal and pediatric congenital vascular diseases at 1.5T and 3.0T: comparison of an intravascular contrast agent (Gadofosveset) with an extracellular agent (Gadopentetate Dimeglumine)

**DOI:** 10.1186/1532-429X-15-S1-W32

**Published:** 2013-01-30

**Authors:** SN Khan, C DaSilva, P Renella, G Satou, I Ayad, S Patel, I Boechat, P Finn

**Affiliations:** 1 Radiology, UCLA, Los Angeles, CA, USA; 2 Pediatric Cardiology, UCLA, Los Angeles, CA, USA; 3 Anesthesia, UCLA, Los Angeles, CA, USA

## Background

Compare gadofosveset (Ablavar, Lantheus Medical) at a dose of 0.06 mmol /kg with gadopentetate dimeglumine (Magnevist, Bayer-Schering Inc.) at a dose of 0.2 mmol /kg for CEMRA in pediatric patients with complex congenital heart disease (CCHD) at 1.5T and 3.0T.

## Methods

Twenty-eight pediatric patients with CCHD underwent CEMRA at 3.0T (n=16) or at 1.5T (n=12). Sixteen patients were imaged with gadofosveset; 9 at 3.0T (age 1.00 ± 1.58 months; weight 2.38 ± 1.13 kg) and 7 at 1.5T (age 8.00 ± 7.83 months; weight 5.06 ± 3.09 kg). Twelve patients were imaged with gadopentetate; 7 at 3.0T (age 1.00 ± 1.41 months; weight 3.02 ± 1.59 kg) and 5 at 1.5T (age 6.60 ± 8.62 months; weight 5.23 ± 2.93 kg). High resolution CEMRA was performed in two phases with strictly comparable imaging parameters, acquisition times and contrast agent infusion periods. Two independent observers scored the studies blindly on a four point scale for image quality, artifacts and vessel definition.

## Results

At 3.0T, overall image quality (IQ) was good to excellent (3<=IQ<=4) in all patients and similar for gadofosveset and gadopentate. At 1.5T, IQ was also good to excellent, but higher for gadofosveset than gadopentetate. Cardiac motion or pulsation artifact was found in all studies at both field strengths but appeared more severe at 3.0T than 1.5T. Parallel acquisition artifact was noted in all studies at 3.0T, but in no studies at 1.5T. Vessel definition scores were higher for gadofosveset than gadopentetate in the venous phases at 3.0T and 1.5T. SNR and CNR was higher at 3.0T than 1.5T in the aortic arch, pulmonary artery, inferior vena cava and superior vena cava. SNR and CNR was higher for gadofosveset than gadopentetate during the arterial phase in the aortic arch and pulmonary artery.

**Figure 1:**
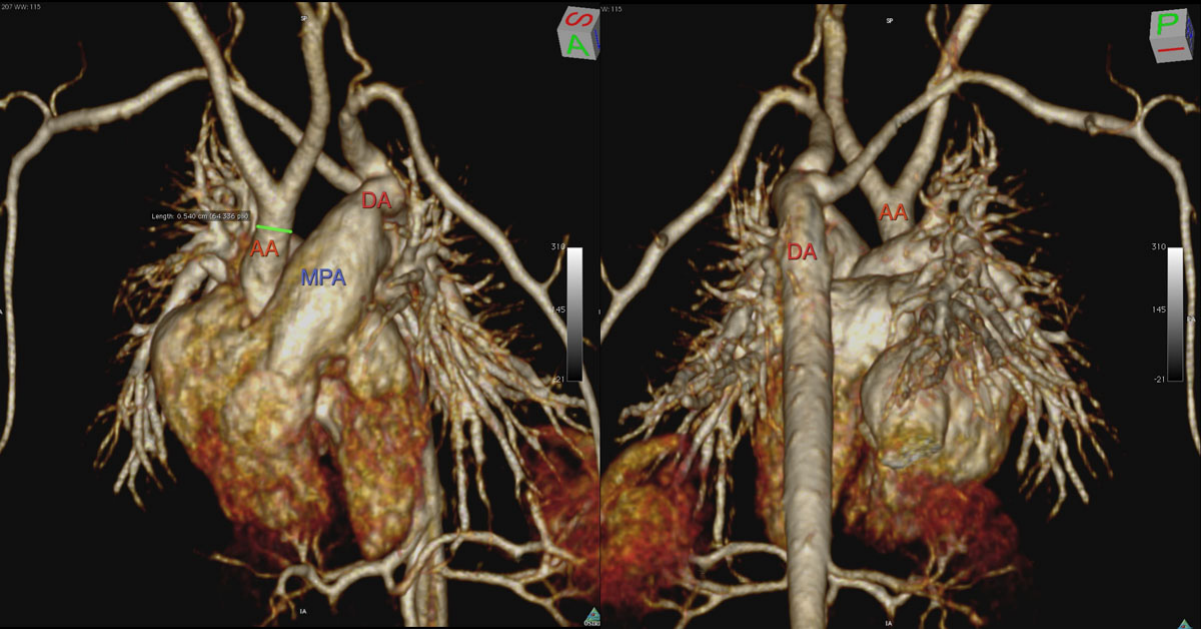
4 day old neonate, 2.27kg, with interrupted aortic arch, large patent ductus arteriosus, large atrial and ventricular septal defects, aberrant right subclavian artery. MR performed at 3.0T with gadofosveset. Volume rendered reconstruction anterior view (left) with hypoplastic ascending aorta and posterior view (right) with interrupted aortic arch.

## Conclusions

Both gadofosveset and gadopentetate support reliable and high quality CEMRA at 1.5T and 3.0T in children with complex congenital vascular diseases. However, gadofosveset scored higher than gadopentetate with respect to image quality, vessel definition, SNR and CNR at less than one third the dose of Gadolinium. Furthermore, the prolonged intravascular residence time of gadofosveset enabled the maximum duration of breath holding to be halved relative to gadopentetate, while maintaining excellent enhancement in the venous phase.

## Funding

Siemens Research Grant

